# Dynamics of Methane-Consuming Biomes from Wieliczka Formation: Environmental and Enrichment Studies

**DOI:** 10.3390/biology12111420

**Published:** 2023-11-11

**Authors:** Weronika Goraj, Anna Pytlak, Jarosław Grządziel, Anna Gałązka, Zofia Stępniewska, Anna Szafranek-Nakonieczna

**Affiliations:** 1 Department of Biology and Biotechnology of Microorganisms, Faculty of Medicine, The John Paul II Catholic University of Lublin, Str. Konstantynów 1I, 20-708 Lublin, Poland; anna.szafranek-nakonieczna@kul.pl; 2 Institute of Agrophysics, Polish Academy of Sciences, Doświadczalna 4, 20-280 Lublin, Poland; apytlak@ipan.lublin.pl; 3 Department of Agricultural Microbiology, Institute of Soil Science and Plant Cultivation–State Research Institute (IUNG-PIB), Czartoryskich 8, 24-100 Puławy, Poland; jaroslaw.grzadziel@gmail.com (J.G.); agalazka@iung.pulawy.pl (A.G.); 4 Department of Biochemistry and Environmental Chemistry, The John Paul II Catholic University of Lublin, Konstantynów 1 I, 20-708 Lublin, Poland; stepz@kul.pl

**Keywords:** enrichment culture, methane, methanotrophs, *Methylomonas*, salt mine

## Abstract

**Simple Summary:**

Deep subsurfaces such as caves and mines are extreme environments inhabited by specialized microorganisms that are able to cope with the adverse conditions caused by low moisture, and high pressure. Our research confirmed that one such site is the rocks surrounding the salt beds in the Wieliczka Salt Mine, where methane-oxidizing bacteria are present. We demonstrated methanotrophic activity in both natural and mineral-supplemented rock material. In the next stage of our research, we confirmed that the methanotrophic community can be cultured on a mineral substrate, using methane as the sole source of carbon and energy source. The issues addressed in this study contribute to our understanding of the biodiversity of microorganisms inhabiting the extreme subsurface biosphere but also provide insights into changes in biodiversity during the isolation of bacterial communities from this type of ecosystem. This information can be valuable for the acquisition of microorganisms with potential applications in biotechnology.

**Abstract:**

The rocks surrounding Wieliczka salt deposits are an extreme, deep subsurface ecosystem that as we studied previously harbors many microorganisms, including methanotrophs. In the presented research bacterial community structure of the Wieliczka Salt Mine was determined as well as the methanotrophic activity of the natural microbiome. Finally, an enrichment culture of methane-consuming methanotrophs was obtained. The research material used in this study consisted of rocks surrounding salt deposits in the Wieliczka Salt Mine. DNA was extracted directly from the pristine rock material, as well as from rocks incubated in an atmosphere containing methane and mineral medium, and from a methanotrophic enrichment culture from this ecosystem. As a result, the study describes the composition of the microbiome in the rocks surrounding the salt deposits, while also explaining how biodiversity changes during the enrichment culture of the methanotrophic bacterial community. The contribution of methanotrophic bacteria ranged from 2.614% in the environmental sample to 64.696% in the bacterial culture. The methanotrophic enrichment culture was predominantly composed of methanotrophs from the genera *Methylomonas* (48.848%) and *Methylomicrobium* (15.636%) with methane oxidation rates from 3.353 ± 0.105 to 4.200 ± 0.505 µmol CH_4 mL_^−1^ day^−1^.

## 1. Introduction

Microbiological studies conducted beneath the Earth’s surface, with the aim of exploring the biodiversity of the subsurface biosphere, are of great significance as they expand the current scientific framework. Microbial life in extreme environments is influenced by physical, geochemical, and biological factors, including the availability of liquid water, nutrients, trace elements, and pressure. Recent research has demonstrated the presence and activity of microbial cells in the deep biosphere [1,2,3]. Within the microbial communities inhabiting underground extreme environments, groups known as methane-consuming organisms (methanotrophs) represent a small but often functionally important trait.

Phylogenetically, aerobic methanotrophs can be classified into α- or γ-Proteobacteria, and Verrucomicrobia. They have been a subject of interest to scientists for many years. A significant advancement in understanding methanotrophs occurred in 1970 when Whittenbury and colleagues described over 100 new isolates [4]. In general, these organisms utilize methane as a source of both carbon and energy, although recent studies indicate that methanotrophs, especially those belonging to the α-Proteobacteria, also show growth on other substrates [5]. The first methanotrophic organism to have its genome sequenced was *Methylococcus capsulatus* (Bath). Since then, the genomes of numerous methanotrophs have been sequenced, including *Methylosinus trichosporium* OB3b [6], *Methylocella silvestris* BL2 [7], *Methylomonas methanica* MC09 [8], *Methylobacter tundripaludum* SV96 [9], *Methylotuvimicrobium* (previously *Methylomicrobium*) *alcaliphilum* 20Z [10], and *Candidatus Methylacidiphilum infernorum* [11].

Aerobic methanotrophs occur in terrestrial and aquatic environments, where oxygen is available as an electron acceptor and CH_4_ as an energy and carbon source [12]. Methanotrophs have been isolated from various environments, such as marine waters, lagoons, and alkaline lakes, peat bogs [13,14,15,16,17]. Biodiversity analyses of microorganisms inhabiting various extreme environments like coal deposits [1,2,18,19,20], groundwater [21,22,23], and subsurface petroleum reservoirs [24] have shown that methanotrophs are also a component of the subsurface microbiomes. Our previous research revealed that the Wieliczka Formation is the habitat of a methanotrophically active community [25,26,27]. We hypothesize that the rock surrounding the salt beds may serve as a new source of methanotrophic bacteria capable of efficiently degrading methane, thus with potential biotechnological application. Therefore, the objective of this study is to demonstrate the possibility of culturing methanotrophs from these extreme environmental samples. Furthermore, since the diversity of the microbial communities persists in tight relation to methanotrophic activity [28], we analyzed the changes in biodiversity during the enrichment of the salt-related methanotrophic community.

## 2. Materials and Methods

### 2.1. Study Site and Samples Collection

**Figure 1:**
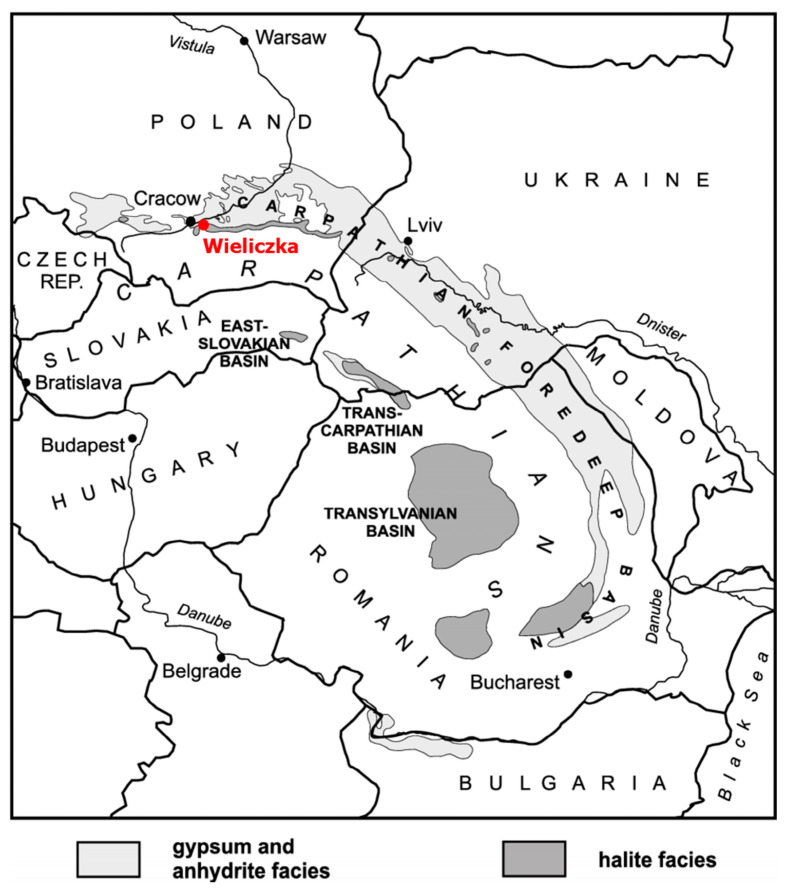
Location of Miocene evaporite deposits in the Carpathian Foredeep (modified after [32]).

The research materials used in this study were rocks from the surroundings of salt deposits in the Wieliczka Salt Mine, specifically siltstones containing veins of fibrous salt and lenses of anhydrite (referred to as W4). These rocks were previously characterized by Stępniewska et al. in 2018 [25]. The Wieliczka Salt Mine is located in southern Poland, approximately 13 km northeast of Kraków, and can be accessed through the mine workings (Figure 1). The mine is situated within the Wieliczka Formation (49°59′ N 020°03′ E), which extends over a length of approximately 10 km and covers an area of the Carpathian Foredeep and the adjacent Carpathian Foreland [29]. The formation’s origin is associated with a salinity crisis that occurred between an estimated 20,000 and 600,000 years ago [30,31]. This crisis led to the disappearance of large marine basins.

The samples (W4) of sedimentary rocks surrounding the salt deposit in the Wieliczka Salt Mine described as siltstone with veins of fibrous salt and lenses of anhydrite were collected at a depth of 130 m. The procedure for collecting samples was outlined in the study conducted by Goraj et al. in 2021. The solid clumps (ca. 4 kg) were extracted from freshly excavated surfaces of Wieliczka Formation sediments, immediately put into sterile plastic containers, and tightly sealed. Samples used for DNA extraction and microbial enrichment were externally sterilized by UV irradiation and flaming as previously described [26,31].

### 2.2. Determination of Methanotrophic Activity of Environmental Sample

The methanotrophic activity of the environmental samples was assessed through a controlled incubation process. Initially, 15 g of crushed rock material was placed inside sterile dark glass bottles, with a volume of 60 mL. The incubation period lasted for 77 days. Two moisture conditions were tested: natural moisture (W4n) and an increased moisture level at 200% of the total water capacity (t.w.c). The latter was achieved by adding sterile degassed water to the samples. Incubations were performed in triplicate in an air atmosphere enriched in CH_4_ at a beginning concentration in the headspace above 10% (*v*/*v*).

To measure changes in methane concentrations over incubation time, a Varian CP-3800 gas chromatograph equipped with a flame ionization detector (FID) at a temperature of 200 °C was used [1,2,33].

### 2.3. Enrichment of Methane-Oxidizing Bacterial Community

Enrichment and culture of methanotrophs were performed on the basis of the environmental sample (W4), whose methanotrophic activity we demonstrated in our previous work [26]. The biologically active environmental sample obtained from this incubation was subsequently used for further laboratory analyses. The conditions for the preparation of all samples processed at the various stages of isolation of the methanotrophic enrichment culture are presented in Table 1.

**Figure 2:**
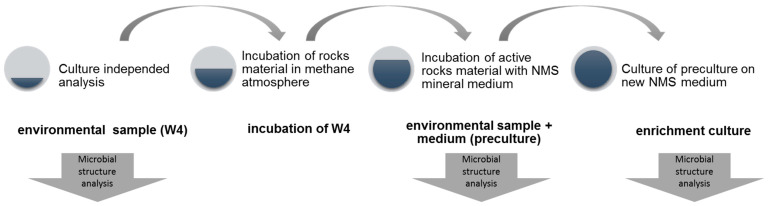
Stages of enrichment of the methane-oxidizing bacteria from environmental material being rocks associated with salt deposits at the Wieliczka Salt Mine.

W4, preculture, and enriched culture samples were selected for further study. The sample described as W4 incubation was an intermediate stage where rock material was incubated in a methane atmosphere to test its ability to oxidize methane (Figure 2).

A culture of methanotrophs on NMS (Nitrogen Mineral Salt) medium, as described by Whittenbury in 1970 [4], was initiated by inoculating the medium with methanotrophically active rock at a ratio of 1:5 (rock to medium). The incubations were carried out in sterile incubation bottles with a volume of 120 mL at a temperature of 30 °C, with constant shaking at 180 rpm, and in an atmosphere containing 10% (*v*/*v*) methane. Once the methanotrophic activity, indicated by methane consumption, was confirmed, 0.1 mL of the culture was transferred to a fresh NMS medium to establish a new culture. This process of transferring the culture to a new medium was repeated three times to obtain the target enrichment culture. Each time, the inoculum (the culture from the previous stage being in the logarithmic growth phase) was 10% of the volume of the new culture.

Throughout the incubation period, the growth of microorganisms in the cultures was monitored by measuring the optical density at 600 nm (OD_600_) using a spectrophotometer (UV-1800, Shimadzu). Additionally, a chromatographic analysis of methane consumption (Varian CP-3800, FID detector) was performed. The methanotrophic activity of the enrichment culture was determined based on the decrease in methane concentration in time and expressed as a µmol of oxidized methane per ml of medium per day of incubation (µmol CH_4 mL_^−1^ day^−1^). The data obtained from these measurements allowed for the determination of growth curves for individual cultures over time.

### 2.4. Bacterial Diversity and Molecular Techniques

The isolation of genomic DNA from samples (environmental sample (W4); environmental sample + medium (preculture) and enrichment culture) followed the procedure described in our previous publication [26]. For this process, rock samples weighing 10 g were aseptically collected from the interior of the clumps. These rock samples were then directly transferred into the beadbeating solution of the DNeasy PowerMax Soil Kit (Qiagen, MD, USA) and further processed according to the manufacturer’s protocol. To extract the total bacterial DNA from the cultures, the Sambrook and Russell method was employed, with their own modification as previously described by [34,35]. Triplicate extractions (from each of these samples) were pooled for downstream analyses. To verify the presence of DNA, electrophoresis was performed using a 1% agarose gel with 1×TBE buffer, and nucleic acid detection was achieved using a SimplySafe™ stain (EURX, Gdańsk, Poland).

The V3-V4 hypervariable regions of the bacterial 16S rRNA gene were amplified using the primer pair: 341F-CCTACGGGNGGCWGCAG and 785R-GACTACHVGGGTATCTAATCC [35]. Each sample was amplified with NEBNext^®^ High-Fidelity 2×PCR Master Mix (New England BioLabs, MA, USA) according to the manufacturer’s instructions. Paired-end (2 × 250 nt) sequencing was performed with an Illumina MiSeq by Genomed S.A. (Warsaw, Poland) and following the manufacturer’s run protocols (Illumina, Inc., San Diego, CA, USA).

### 2.5. Bioinformatic and Statistic Analysis

Bioinformatics analysis was conducted following the methodology outlined in our prior publication [26]. Demultiplexed fastq files were processed using the DADA2 (version 1.12) package [36] in R software (version 3.6.0) [37]. Forward and reverse reads were trimmed to 250 bp, primer sequences were removed from all reads. The filtering parameters were as follows: maxN = 0, maxEE for both reads = 3, truncQ = 2. MaxEE corresponds to the maximum expected errors. Expected errors are calculated from the quality score (EE = sum (10^(−Q/10)^). Sequences were dereplicated using derepFastq with default parameters, and exact amplicon sequence variants (ASV) were resolved using Dada. Next removeBimeraDenovo was used to remove chimeric sequences. After the filtration steps, 93,703–107,115 (mean = 99,208) of the reads were left for further analysis. Taxonomy assignments were made against the latest version of the modified RDP database [38] using IDTAXA [39]. Sequences originating from chloroplast or mitochondrial DNA were excluded from further analysis. For subsequent analyses, the total number of reads for each taxa was converted to percentages, with the sum of all taxa in individual samples assumed to be 100%.

Alpha diversity indices were calculated using the phyloseq package. Hierarchical clustering was performed in the pheatmap package in R [40] and rows and columns were ordered based on the order of hierarchical clustering (using the “complete” method) using the Bray-Curtis distance matrix.

Venn diagrams were generated using an online tool (http://bioinformatics.psb.ugent.be/webtools/Venn/, accessed on 1 October 2023).

### 2.6. Data Availability

NGS sequence data have been deposited in the NCBI Sequence Read Archive (SRA) database under BioProject’s ID: PRJNA596092.

## 3. Results and Discussion

### 3.1. Enrichment and Characterization of a Methane-Oxidizing Bacterial Community

**Figure 3:**
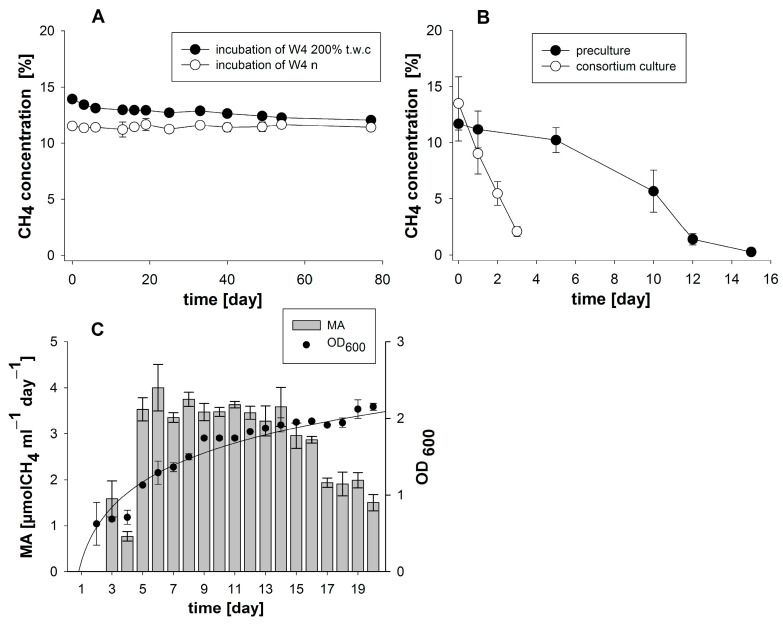
Gas dynamics during incubation of environmental samples (W4) (**A**) and incubation of rock material in NMS medium (preculture) and during the first 3 days of methanotrophic enrichment culture growth (**B**). Growth curve of the enrichment culture and its methanotrophic activity (MA) (**C**).

The dynamics of the change in methane concentration during the incubation of rock material (W4) in two moisture variants are shown in Figure 3A. A decrease in CH_4_ concentration was noted only in variants with 200% t.w.c., indicating that moisture is a limiting factor for methanotrophs activity in the investigated material. At those conditions, the methanotrophic activity (MA) rate was on average 0.220 ± 0.066 micromoles of CH_4_ per gram of dry weight of rocks per day (µmol CH_4_ gdw^−1^ day^−1^) [26].

The second step to obtain a culture of methanotrophic bacteria from the Wieliczka mine was to incubate methanotrophically active rock material in an NMS medium. This enrichment step was termed preculture. During the 15-day incubation period the significant oxidation rate of the added CH_4_, from 11.68 to 0.258% *v*/*v*, was observed (Figure 3B). MA calculated on the basis of measured methane concentration decrease equaled 1.211 ± 0.197 µmol CH_4_ gdw^−1^ day^−1^ and was 5.5 times higher than in full saturated samples from the first stage of the experiment.

The final methanotrophic culture was obtained by passaging the culture four times onto a new medium. On the basis of gas-phase chromatographic analysis during culture, at the final stage of enrichment, almost complete methane consumption was recorded (Figure 3B). Throughout this period, the optical density of the culture showed an increase of about 0.5 units each measurement day (Figure 3C). Subsequent stages of culture necessitated the balancing of gas levels within the incubation vessels. This entailed the replenishment of the methanotrophic substrate-CH_4_, as well as the adjustment of O_2_ and CO_2_ concentrations to match atmospheric levels. As the culture’s microorganisms grew and their CH_4_ requirements intensified, gas concentrations were harmonized every 1–3 days. The outcome yielded a community of microorganisms growing on methane as its sole carbon and energy source. Its MA was ascertained to span from 1.582 ± 0.392 µmol CH_4 mL_^−1^ day^−1^ after 3 days of culture (Figure 3C). Notably, during the exponential growth phase, there was a marked surge in the culture’s optical density (OD_600_), peaking at around 3 OD units. Subsequently, the oscillation of methanotrophic activity ranged between 3.3534 ± 0.1047 and 4.2000 ± 0.5054 µmol CH_4 mL_^−1^ day^−1^ (Figure 3C).

### 3.2. Bacterial Diversity

The combination of previously published findings, which encompass the analysis of physicochemical properties [25], the evaluation of methane oxidation capacity [26], and the metataxonomic analysis of the microbial community [26], along with the outcomes derived from the assessment of methanotrophic activity during various stages of the enrichment culture separation process in this study, collectively provide substantiated evidence for the potential existence of methanotrophic microorganisms within rocks linked to salt deposits in the Wieliczka Salt Mine.

To achieve a comprehensive understanding of microbial diversity, we employed next-generation sequencing (NGS).

At each successive enrichment step, a decrease in culture biodiversity was found among the entire Bacteria domain as well as quantitative and qualitative changes within the methanotrophs. During enrichment cultures, there was an increase in the percentage of methanotrophic and methylotrophic microorganisms.

**Figure 4:**
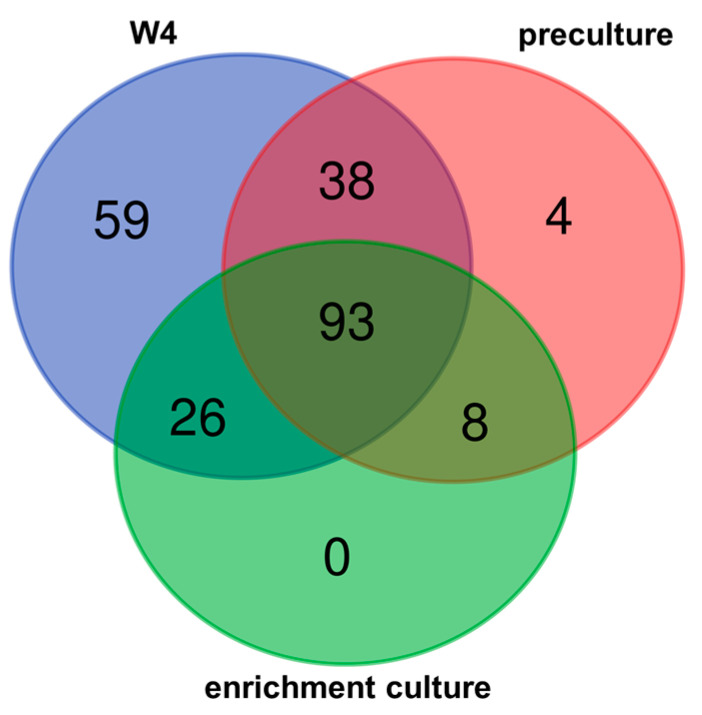
Venn diagram showing the number of genera specific to a given sample and common to all samples.

The greatest biodiversity was demonstrated in the environmental sample, where among 20 phyla and 43 classes, 216 genera were identified. On the other hand, metataxonomic analysis of enrichment culture showed the presence of bacteria classified into 16 phyla, 26 classes, and 127 genera. The decline in biodiversity is also reflected in the values of the biodiversity indices. In the environmental sample, the H-index value was 3.064, in the sample enriched with NMS medium it was 2.665, while the methanotrophic culture had a Shannon-Wiener index of 0.931. All genera identified in the enrichment culture were also present in the environmental sample. Furthermore, 93 taxa were found to be common to all enrichment stages (Figure 4).

Information on microorganisms inhabiting the rocks surrounding salt deposits available in the literature is scarce. However, the first to isolate microorganisms from salt deposits were Dombrowski [41] and Tasch [42]. Contemporary examples of research related to the microbiology of salt mines include work related to the identification of microorganisms from salt mines in Pakistan [43,44,45], India [46], Israel [47], and China [48,49]. Due to the economic importance of the energy industry in recent years, one of the dominant branches of geomicrobiology has been associated with coal and lignite deposits. These environments host diverse microbial communities, the composition of which also includes methanotrophic bacteria [18,50,51].

Methanotrophs identified in rocks surrounding Badenian salts at Wieliczka (*Methylomicrobium*, *Methylcysteace*) have also been described in older geological formations found in Poland. Wolińska and coworkers [1] studied methane-related microorganisms inhabiting the Upper Silesian coal Basin (USCB) located in southern Poland. They showed that these bacteria belonged to the genera: *Methylosinus*, *Methylobacter*, and *Methylocystis* [1]. Similar research, but in the Lublin Coal Basin, was conducted by Stępniewska et al. [18]. At that time it was shown that the rocks of Carboniferous coal seams contain methanotrophic bacteria of the genera: *Methylosinus*, *Methylomicrobium*, *Methylcystis,* and *Methylocaldum* [18].

### 3.3. Changing Biodiversity of the Methanotrophic Community

**Figure 5:**
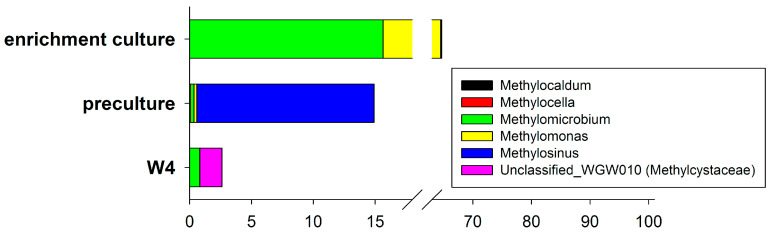
Qualitative and quantitative changes in the structure of methanotrophs during enrichment culture of the methanotrophic community from rocks accompanying salt beds.

The purpose of the study was the enrichment and cultivation of a methane-oxidizing community. We found that the relative abundance of CH_4_-utilizing microorganisms has changed from 2.614% in the environmental sample to as much as 64.696% in the final culture (Figure 5).

In an environmental sample, *Methylomicrobium* spp. (0.817%) and Unclassified_WGW010 (1.797%) were the dominant methanotrophs. Regarding Unclassified_WGW010, this cluster was identified as representing methanotrophic bacteria since it exhibits 100% identity with uncultured *Methylocystaceae* (EF072118.1) [26].

The structure of the methanotrophic microbiome in the enrichment culture (consisting of the environmental sample and medium–II stage of enrichment) revealed a significant decrease in the relative abundance of the genus *Methylomicrobium* by over 72% compared to its abundance in the original environmental sample. Interestingly, the dominant methanotrophs in the enriched culture were identified as *Methylosinus*, constituting 14.339% of the total community.

At the final stage of methane-oxidizing bacteria enrichment, the distribution of individual methanotrophic genera displayed notable differences. The dominant genus was *Methylomonas* (48.848%) and among the associated methanotrophs *Methylomicrobium* (15.636%) and *Methylosinus* (0.194%) were found. In addition to the dominant and associated genera, there were two other methanotrophic taxa, *Methylocaldum* and *Methylocella*, which were present but at a considerably lower relative abundance of less than 0.05%. (Figure 5).

The varying proportions of the identified genera suggest that different methanotrophs show differential responses to enrichment conditions. Also significant are the complex relationships both between methanotrophs and heterotrophs and between different genera of methanotrophs. As a result, the composition of the enrichment culture at particular points in the cultivation process in the final isolation phase is a result of many factors. As shown by other authors, the enriched methanotrophic culture can be dominated by *Methylomonas* sp. or *Methylosinus* sp. depending on the culture period [52]. *Methylosinus* is a Type II methanotroph whose abundance is promoted by methane and copper-limiting conditions [29]. Thus, the larger proportion of *Methylosinus* in the environmental sample enriched with culture medium could be related to methane limitation at the end of the incubation process. Differences in the relative abundance Unclassified_WGW010, classified within the Methylcystaceae family, and the genus *Methylomonas* between environmental and enrichment samples appear to be logical. Within the Methylocystaceae family, the genus *Methylocystis* is recognized as an oligotroph and a facultative type II methanotroph [53]. Its adaptable metabolic capabilities enable it to utilize alternative simple carbon sources like acetate, providing a potential advantage over copiotrophic counterparts such as *Methylomonas* [30]. A study conducted by Valverde-Pérez and coworkers highlighted that *Methylomonas* tends to dominate when the proportion of *Methylcystis* is very low, and conversely, *Methylcystis* dominates when the presence of *Methylomonas* is limited [30]. This highlights the complex relationship and potential competition between the two genera and, as a result, accounts for the composition of a given methanotrophic consortium.

The prevalence of *Methylomonas* as the dominant genus suggests its advantageous adaptation to the specific enrichment conditions and the medium used during the isolation process. The varying proportions of the identified genera imply that different methanotrophs respond differently to the enrichment conditions, resulting in a unique composition at the final stage of enrichment culture. Understanding the dynamics and interactions among these methanotrophic genera and between methanotrophs and other bacteria is crucial for gaining insights into their roles in methane metabolism and their potential contributions to environmental processes.

### 3.4. Enrichment Culture Composition

The enrichment culture was dominated by Proteobacteria (80.85%) but Bacteroidetes (11.36%) and Verrucomicrobia (7.46%) were also reported. Both Proteobacteria and Verrucomicrobia include methanotrophic bacteria. Representatives of the Bacteroidetes, on the other hand, can function in a cultured community as companion microorganisms to the methanotrophs. The literature provides examples of Bacteroidetes that have been identified in methane-rich environments [31,54,55], but to date, there are no data confirming the ability of Bacteroidetes to oxidize methane. However, it has been confirmed that the presence of these bacteria contributes to the methanotrophic activity of the entire microbial community, which may be due to the phenomenon of cross-feeding (“cross-feeding”). It means that products produced by methanotrophs during methane oxidation are a substrate for heterotrophic Bacteroidetes [33,34,35].

The genus *Methylomonas*, which was the dominant component of the enrichment culture from the Wieliczka Formation (almost 49%, Figure 2), belongs to type I methanotrophs. They are mesophiles, show optimum growth at 25–30 °C and most species are halotolerant [56]. To date, they have been found in various ecosystems including lake and river sediments [57,58,59,60,61], seawater [8], peatlands and other wetlands [62,63,64,65], wastewater, groundwater, and coal mines [56], and organic-mineral sediments from copper mines [66]. The diversity of environments from which *Methylomonas* spp. have been isolated so far and their ability to survive in extreme conditions justify their occurrence in the studied environmental sample and consortium. In addition to *Methylomonas*, the enrichment culture included bacteria of the genus *Methylomicrobium* (more than 15%). They are mostly mesophilic microorganisms, with some strains being alkalotolerant or alkalophilic (pH 9–10.5), as well as halophilic. Like the dominant genus *Methylomonas* in the enrichment culture, *Methylomicrobium* spp. has been found in various ecosystems, such as sediments, lake and river waters, soils, peatlands, and ocean waters [67,68,69].

**Figure 6:**
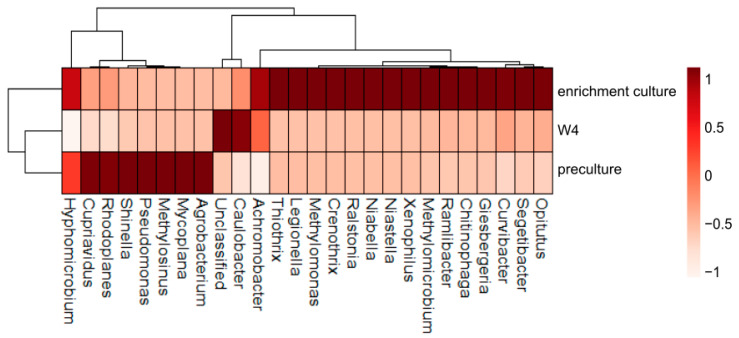
The taxonomic composition of the methanotrophic enrichment culture juxtaposed with the original environmental material (W4) and the preculture. The bacterial genera depicted in the presentation account for over 0.1% of the entire bacterial community within the enrichment culture. The heatmap presents normalized data.

The microorganisms associated with the methanotrophs in the enrichment culture, whose percentage was above 0.1% among all identified genera, were also mostly present in situ in the environmental sample (Figure 6).

In addition to methanotrophs, a noteworthy component of the final enrichment culture was bacteria from the genus *Niabella*, constituting approximately 9% of the total identified population. These bacteria belong to the chemoorganotrophs and exhibit mesophilic, halotolerant, and halophilic aerobic characteristics [70]. To date, bacteria belonging to the *Niabella* genus have been observed within diverse ecosystems. They were identified as part of a consortium involved in rice straw decomposition [71], a thermophilic cellulose hydrolyzing consortium [72], and have been found to form biofilms in recirculating aquaculture systems or inhabit methane biofilters [73]. Significantly, it is worth noting that the prevalence of this genus was scarce in the environmental sample, constituting merely 0.0007% of the population, and in the NMS-enriched sample, it contributed to 0.005% of the overall readings obtained (Figure 3). *Opitutus* accounted for almost 7% of the enrichment culture community and their percentage was increasing. In situ identification in rock material indicated 0.975% and in incubation enriched with NMS medium 0.002% share of this genus among all recognized bacterial genera. Bacteria of the genus *Xenophilus*, which initially also accounted for a small percentage of 0.0004–0.0007%, eventually accounted for 2.379% of the taxonomic composition of the studied methane-oxidizing bacterial community (Figure 6). The literature provides several examples of the occurrence of *Xenophilus* spp. in the environment [74,75,76]. In addition, the proportion of bacteria of the genus *Pseudomonas* increased by 30.481% already at the first stage of isolation, and as a result, they occurred at 1.983% in the enrichment culture (Figure 6). The proportion of *Hyphomicrobium* spp. increased from 0.116 in the environmental sample and 0.971 in the medium-enriched sample to 1.290% in the enrichment culture. A similar trend was observed for the genus *Crenothrix*, for which there was an increase with respect to the environmental sample of 0.007% in the first stage of culture and 1.089% in the final culture. On the other hand, bacteria of the genus *Cupriavidus* in the environment were present at 0.897%, after enrichment with the medium they accounted for 1.811% of all identified genera, and finally, in the enrichment culture, their percentage was set at 1.098% (Figure 6).

Bacteria that do not have the ability to oxidize methane, occurring when cultured on a mineral medium in an atmosphere containing methane, have significant importance and impact on the functioning of the entire enrichment culture. Firstly, as mentioned earlier, by-products produced by methanotrophs during methane oxidation can serve as their substrates. It can also be assumed that interactions in the trophic network of the enrichment culture community rely on dead methanotrophs as food for heterotrophs [77]. Heterotrophs may also provide a source of vitamins for methanotrophs or mediate interspecies electron transfer that enhances community metabolism [78]. Ho et al. [28] found that the growth rate and methane oxidation capacity of methanotrophs gradually increased with increasing heterotrophic microorganism diversity.

### 3.5. The Importance of Microorganism Community in Biotechnology

The results of the present study indicate the feasibility of obtaining a consortium capable of aerobic oxidation of methane. Microorganism communities and consortia hold immense significance in the realm of biotechnology due to their multifaceted capabilities and applications. In essence, microorganism consortia represent a versatile tool in biotechnology, offering solutions to various challenges across environmental, industrial, and agricultural sectors [79,80].

Consortia often consist of different microbes, each contributing specialized functions. This diversity allows them to perform a wide range of tasks, from metabolizing complex substrates to producing valuable compounds. Microbial consortia can exhibit syntrophic interactions where the activities of one species support or enhance the growth and functions of others. An example of syntrophic interaction is the coexistence of two genera of methanotrophs *Methylomonas* and *Methylophilus* in consortia, which has been confirmed in recent studies [52,81,82]. The benefits of bacterial consortia of methanotrophs and heterotrophs have been noted, for example, in the production of microbial proteins by methanotrophs [30,83]. Other researchers have demonstrated that consortia are more efficient than pure cultures in the production of valuable compounds e.g., polymers (PHAs) [84,85] or ectoine [86]. The presence of heterotrophs ensures culture stabilization by consuming potentially inhibitory metabolic byproducts or contaminants [87]. A huge advantage of using consortia is that they do not require sterile conditions and have more favorable nutritional requirements [88]. Moreover, mixed methane-oxidized consortia can adapt to complex environments and have higher application potential [20]. Thus, synergistic interactions between organisms in the environment provide attractive advantages that can be implemented in various biotechnological processes. Nevertheless, it should be remembered that it is very important to ensure the safety of microbial consortia by excluding the presence of potential pathogens in the composition of these communities.

Noteworthy, the use of advanced molecular techniques such as high-throughput sequencing provides a valuable tool to help enrich and isolate consortia from the environment and characterize them. Lau et al. (2015) employed sequencing of 16S rRNA gene amplicon regions to characterize methanotrophs affiliated with the Methylococcaceae and Methylocystaceae families across diverse ecosystems (including forest soil, peat soil, and *Sphagnum* moss) [89]. Despite methanotrophic bacteria constituting less than 2% of the microflora within these environments, the study identified a total of 63 operational taxonomic units (OTUs) associated with the aforementioned families. Furthermore, specific genes related to methanotrophy, such as *pmoA* genes, can also undergo sequencing. Sengupta and Dick (2017) employed this approach to investigate the diversity of methanotrophs in two distinct soil habitats [90].

## 4. Conclusions

The presented results show that the methanotrophically active W4 rock surrounding salt deposits in the Wieliczka Salt Mine can serve as a new source of methanotrophic bacteria capable of efficient methane degradation. The discrepancy in the representation of various methanotrophs between the rock material and the enriched culture results from the culture conditions provided in the presented experiment as well as the interdependencies between individual microorganisms in the culture. These factors drive the selection and dominance of specific microbial groups during enrichment processes. The research we propose is of a model nature (“from the environment to the bacterial culture”), allowing for a quick check of the possibility of culture methanotrophs from a difficult matrix, such as the extreme environment of the Wieliczka Formation. Moreover, the potential possibility of using the biotechnological potential of the isolated community for methane oxidation was demonstrated.

## Figures and Tables

**Table 1:** Description of the samples prepared at the various stages of the separation of the methanotrophic enrichment culture.

Sample Name	Research Material	Medium	Substrate Addition (CH_4_)
W4	crushed rocks surrounding Wieliczka salt deposits	no medium	no CH_4_
W4 incubation	crushed rocks surrounding Wieliczka salt deposits	destiled water	approx. 10% CH_4_
preculture	W4 incubation	mineral medium (NMS)	approx. 10% CH_4_
enrichment culture	preculture	mineral medium (NMS)	approx. 10% CH_4_

## Data Availability

NGS sequence data have been deposited in the NCBI Sequence Read Archive (SRA) database un-der BioProject’s ID: PRJNA596092 (https://www.ncbi.nlm.nih.gov/bioproject/?term=PRJNA596092, accessed on 1 October 2023).
